# Editorial: Children and Companion Animals: Psychosocial, Medical and Neurobiological Implications

**DOI:** 10.3389/fvets.2018.00112

**Published:** 2018-06-04

**Authors:** Andrea Beetz, Lynette Arnason Hart, Brinda India Jegatheesan, Naoko Koda

**Affiliations:** ^1^Department of Special Education, University of Rostock, Rostock, Germany; ^2^Department of Population Health and Reproduction, School of Veterinary Medicine, UC Davis School of Veterinary Medicine, Davis, CA, United States; ^3^College of Education, University of Washington, Seattle, WA, United States; ^4^School of Agriculture, Tokyo University of Agriculture and Technology, Fuchu, Japan

**Keywords:** children, companion animals, human-animal interaction, animal-assisted intervention, animal-rearing, autism, dog bites, anthrozoology

**Editorial on the Research Topic**

**Children and Companion Animals: Psychosocial, Medical, and Neurobiological Implications**

This Research Topic presents experiences with companion animals provided to children in varied settings. Methods include monitoring of the ongoing interaction of children with companion animals, or conducting interviews or surveys, as well as experimental interventions where changes occurring with the presence of the animal are assessed. Children in Austria, Germany, Jamaica, Japan, and the United Kingdom, as well as a minority from the United States, are represented in these studies.

## Animals in Educational Contexts

Nakajima contrasts two approaches: incorporating animals into school curricula or employing them in interventions to facilitate specific learning. Animal-rearing is a Japanese educational tradition, specified by the Japanese government in the course of study for formal elementary education. Matsuda (1, 2), a science educator, laid the theory and methods showing the value for children of rearing animals; it thus became a method of teaching. “Education through assisting animals,” embeds animal-rearing within formal education as an educational tool.

Koda et al. describe how some classrooms integrate rearing goats into instruction at urban schools in Japan. Children share responsibility for feeding, cleaning, and caring for the goats, while working with teachers. Teachers view experience with goats as beneficial to the children. Challenges during holidays and weekends require cooperation among faculty and parents. Some teachers would be cautious about recommending goat-keeping to other schools unless they were very well-prepared.

More conventionally for animal-assisted education, Schretzmayer et al. tested an intervention using a dog to facilitate reading performance and physiological relaxation of third graders lacking reading proficiency. Although a calming effect of the dogs might have been expected due to an oxytocin effect (3), the presence of a dog was associated only with short-term improvements in reading performance, and small changes in cortisol and behavior, for these children with low reading skills.

## Pet Animals for Children

Although most exposure of children to pets is at home, exactly how children interact with the pets at home is unknown. Westgarth et al. employed a large database to assess dog-walking by children up to 18 years of age in the United Kingdom. Simply having a dog was not associated with more walking by the child. Previously, it might have been assumed that children would naturally engage in walking their dogs.

Having a dog at home presents some level of risk of dog bites to children. Arhant et al. conducted a web survey of 402 caregivers living with a dog and a child up to 6 years of age. Aggressive growls but not bites were reported, and only non-serious injuries were generally associated with the child giving the dog treats or taking objects from the dog during play. Messam et al. focused specifically on dog bites to children 5–15 years of age, with 297 interviews of parents in Kingston, Jamaica, and San Francisco. Where the dog slept, and whether the dog had access to a yard, played a role in the likelihood of the child having a dog bite; patterns of dog ownership and dog bites differed between the two cities. Younger children, and especially boys (10.7 and 12.0% in San Francisco and Kingston, respectively), experienced the most bites.

For children diagnosed with Autism Spectrum Disorder, interest is growing in the possible use and value of assistance dogs (4), throughout the world (5). Currently, no method assists parents of autistic children in pet selection; they may just acquire more than one. Guérin et al. argue for the importance of offering choices to children with autism who may benefit from contact with animals, even those who are profoundly affected and non-verbal. They propose developing electronic presentations of active animals so the autistic child can witness the animal and its behavior, and reveal their level of interest in a particular animal.

Families having both a companion cat and an autistic child 3–12 years of age participated in a study by Hart et al. to explore the extent to which cats are compatible pets with these children. The cats exhibited affectionate behavior and very low levels of aggression with the children. The children were very attracted to the cats, pointing to the value of selecting well-socialized cats for autistic children.

## Next Directions

A broad range of topics on children and pets remains unexplored, including cross-cultural ways of dealing with children’s specific challenges such as autism (6). Positive health effects of animal ownership on children (such as on allergies, obesity, immune system) should be addressed in future research. Also, in animal assisted education, many research questions are open, e.g., effects of school dogs on children of different ages, with and without special education needs. Research also is needed on underlying mechanisms of positive effects of human-animal interaction, e.g., activation of the oxytocin system; results so far pertain to adults, and should be replicated with children, if non-invasive methods are available today.

## Author Contributions

All authors listed have made substantial, direct, and intellectual contributions to the work and approved it for publication.

## Conflict of Interest Statement

The authors declare that the research was conducted in the absence of any commercial or financial relationships that could be construed as a potential conflict of interest.

The handling Editor declared a shared affiliation, though no other collaboration, with one of the authors LH.

## References

[1] Matsuda R Practical teaching study at teaching department workshop of teachers’ school held by the Ministry of Education: the first section: teaching of fifth grad science at elementary school. Educ Study (1909) 64:35–41.

[2] Matsuda R The Newest Science Teaching Method. Tokyo: Ryomeido Shoten (1911).

[3] Beetz A Uvnäs-Moberg K Julius H Kotrschal K Psychosocial and psychophysiological effects of human-animal interactions: the possible role of oxytocin. Front Psychol (2012) 3:234 10.3389/fpsyg.2012.00234 22866043 PMC3408111

[4] O'Haire ME Animal-assisted intervention for autism spectrum disorder: a systematic literature review. J Autism Dev Disord (2013) 43(7):1601–22. doi 10.1007/s10803-012-1707-5 23124442

[5] Walther S Yamamoto M Thigpen AP Garcia A Willits NH Hart LA Assistance dogs: historic Patterns and roles of dogs placed by ADI or IGDF accredited facilities and by Non-Accredited U.S. Facilities. Front Vet Sci (2017) 4:1 10.3389/fvets.2017.00001 28154816 PMC5243836

[6] Jegatheesan B Cross-cultural issues in parent professional interactions: A qualitative study of perceptions of Asian American mothers of children with developmental disabilities. Res Pract Persons Severe Disabl (2009) 34(3):123–36. 10.2511/rpsd.34.3-4.123

